# Synergistic Antimicrobial Interaction between Honey and Phage against *Escherichia coli* Biofilms

**DOI:** 10.3389/fmicb.2017.02407

**Published:** 2017-12-08

**Authors:** Ana Oliveira, Henrique G. Ribeiro, Ana C. Silva, Maria D. Silva, Jessica C. Sousa, Célia F. Rodrigues, Luís D. R. Melo, Ana F. Henriques, Sanna Sillankorva

**Affiliations:** LIBRO – Laboratório de Investigação em Biofilmes Rosário Oliveira, Centre of Biological Engineering, University of Minho, Braga, Portugal

**Keywords:** *E. coli*, honey, bacteriophage, biofilms, synergy

## Abstract

Chronic wounds afford a hostile environment of damaged tissues that allow bacterial proliferation and further wound colonization. *Escherichia coli* is among the most common colonizers of infected wounds and it is a prolific biofilm former. Living in biofilm communities, cells are protected, become more difficult to control and eradicate, and less susceptible to antibiotic therapy. This work presents insights into the proceedings triggering *E. coli* biofilm control with phage, honey, and their combination, achieved through standard antimicrobial activity assays, zeta potential and flow cytometry studies and further visual insights sought by scanning electron microscopy and transmission electron microscopy. Two Portuguese honeys (PF2 and U3) with different floral origin and an *E. coli*-specific phage (EC3a), possessing depolymerase activity, were tested against 24- and 48-h-old biofilms. Synergic and additive effects were perceived in some phage–honey experiments. Combined therapy prompted similar phenomena in biofilm cells, visualized by electron microscopy, as the individual treatments. Honey caused minor membrane perturbations to complete collapse and consequent discharge of cytoplasmic content, and phage completely destroyed cells leaving only vesicle-like structures and debris. Our experiments show that the addition of phage to low honey concentrations is advantageous, and that even fourfold diluted honey combined with phage, presents no loss of antibacterial activity toward *E. coli*. Portuguese honeys possess excellent antibiofilm activity and may be potential alternative therapeutic agents in biofilm-related wound infection. Furthermore, to our knowledge this is the first study that assessed the impacts of phage–honey combinations in bacterial cells. The synergistic effect obtained was shown to be promising, since the antiviral effect of honey limits the emergence of phage resistant phenotypes.

## Introduction

Chronic wounds take months, years or may even never heal and present a major biological and financial problem on both individual patients and the broader health system. All chronic wounds show a diversified microflora in the deep dermal tissues where the main prevalent populations evident include *Staphylococcus, Escherichia coli, Pseudomonas, Enterobacter, Stenotrophomonas, Streptococcus*, and *Serratia* among other (Petkovšek et al., 2009; Price et al., 2009; Han et al., 2011; Rhoads et al., 2012). Furthermore, the ability of these microorganisms to colonize and form biofilms is one of the main healing obstacles. Biofilms are structured communities of bacterial cells enclosed in a self-produced polymeric matrix and adhered to an inert or living surface (Costerton et al., 1999). Biofilms are formed in a sequential cycle of discrete and well-regulated events starting from: (i) adsorption of macro and smaller molecules to surfaces; (ii) bacterial adhesion to the wound surface and expression of extracellular polymeric substances (EPS); (iii) microcolony formation and biofilm maturation. Cell aggregation in these biofilm communities are well known to block antibiotics from reaching bacteria and also block host’s immune cells contrarily to their planktonic counterparts which lack structure and are not surrounded by a polymeric matrix (Costerton and Lewandowski, 1995; Stewart and William Costerton, 2001; Fux et al., 2005). Occasionally, clusters of biofilms detach from the biofilm structure and start biofilm formation in new sites (Costerton et al., 1999). In wounds, the biofilm matrix, consisting of both EPS and host-derived matrix (e.g., fibrin and collagen), undeniably has influence in the penetration of certain antibiotics. This is mainly due to the overall negative net charge of the matrix that can sequester for instance the positively charged antibiotic tobramycin while readily allowing neutral antibiotics to penetrate the biofilm (Tseng et al., 2013). Besides protection to antimicrobials and host defenses, the biofilm mode of growth confers protection to the microorganism from mechanical and shear forces (McCarty et al., 2012) and also from altered pH, osmolarity, and nutrient limitation (Costerton et al., 1999; Fux et al., 2005).

Honey is a complex substance made up of hundreds of different compounds. Honey’s antimicrobial activity was initially attributed to the high sugar content and low pH and later to the activity of glucose oxidase which catalyses glucose to form hydrogen peroxide and gluconic acid (Henriques et al., 2005; Mandal and Mandal, 2011; Maddocks and Jenkins, 2013; McLoone et al., 2016). In 1988, it was found that some honeys, mainly derived from the manuka shrub (*Leptospermum scoparium*) still retained their antimicrobial activity when catalase was added (inactivating hydrogen peroxide) to the diluted honey (Allen et al., 1991). This non-peroxide antimicrobial activity of Manuka honey has been attributed to the presence of methylglyoxal (MGO), an α-oxoaldehyde that reacts with important biological molecules such as RNA, DNA and proteins (Kalapos, 1999; Blair et al., 2009; Majtan et al., 2012). MGO is a product of the acidic breakdown of dihydroxyacetone (DHA) which is set off by the addition of glucose oxidase by bees while processing honey (Van Eaton, 2014). Honey has a broad spectrum antibacterial activity against bacteria (Cooper et al., 1999) and its high viscosity provides a protective barrier against infections being suitable for skin care, promoting the wound healing, tissue regeneration and anti-inflammatory process (Mandal and Mandal, 2011; Belcher, 2012).

Before the discovery of modern antibiotics, bacteriophages (phages) that are bacterial viruses, and bee hive products such as honey were extensively used for their antimicrobial properties. In fact, the use of honey dates back at 4,000 years, to Ancient Egypt where it was used for the treatment of wounds among other conditions (Cooper, 2005). On the other hand, phages were widely used in the 1920s and 1930s by physicians whom successfully treated a variety of infections. Even after the widespread of antibiotics, phage therapy continued in many countries, such as Georgia, Poland, and Russia (Kutateladze and Adamia, 2010).

Phages are harmless to mammalian cells and are specific for a target bacteria, therefore do not affect the commensal microflora (Hanlon, 2007; Azeredo and Sutherland, 2008; Kutateladze and Adamia, 2010). In contrast with antibiotics, phages have the ability to self-replicate as long as the host is present which implies that a single dose is sufficient. There is, however, an overall lack of studies comparing the effectiveness of phage products with the standard wound care treatments. The few reports assessing the effectiveness of phages and antibiotics on wounds show that postoperative wound infections in cancer patients (Kochetkova et al., 1989) and also postsurgical wounds (Sakandelidze and Meipariani, 1974) had a higher healing success with phage. In a recent study, a commercial preparation of staphylococcal phage Sb-1 was used in patients with diabetic toe ulcers after poor response to conventional therapy (Fish et al., 2016). Ulcers of all patients treated with phage healed, and no adverse effect, tissue breakdown or recurrence of infection were observed. This reinforces one of the major attractive features that has resulted in phage comeback as potential alternatives to commonly used antimicrobials which is the fact that they are effective against antibiotic resistant bacteria (Sillankorva and Azeredo, 2014). Another attractive characteristic is that, although phages interaction with biofilm populations is not thoroughly studied, many *in vitro* reports acknowledge that phages can destroy, to varying extent, mono and mixed biofilm populations (Doolittle et al., 1995; Pires et al., 2011; Melo et al., 2016).

Although modern antibiotics use has meant a decrease in mortality, its widespread use has led to the emergence of antibiotic-resistant bacteria decreasing the treatment options (Cooper, 2005). Considering wound therapy in particular, there are currently no guidelines for patients with infected wounds. This frequently leads clinicians to prescribe antibiotic therapy until healing occurs even if there is no evidence that supports this practice. This misuse in patients with infected and many times even uninfected wounds eventually lead to antibiotic-resistant infections. The European Wound Management Association and the British Society for Antimicrobial Chemotherapy have recently assembled the principles of wound management, antibiotic treatment, and stewardship to provide practical guidance (Lipsky et al., 2016). In this publication, the authors strongly advise to avoid using antibiotics topically for treating wound infections. The efficiency denoted by the use of honey and phages alone against antibiotic-resistant bacteria and biofilms has raised significant interest for topic use in infected wounds. Manuka honey dressings, gel and ointments are commercially available for topical application in human and pet wounds, and studies have shown that its antimicrobial properties accelerate chronic wound healing (Jull et al., 2007; Gethin and Cowman, 2008; Robson et al., 2009). Also, *in vivo* phage studies in rat and pig models suggest that phages are an effective treatment of cutaneous infected wounds (Mendes et al., 2013). A physician-initiated FDA-approved phase I safety trial of phage therapy against skin ulcerations was completed already in 2008 and showed that phages caused no side effects on the patients (Rhoads et al., 2009). Furthermore, PhagoBioDerm (PolymerPharm, Georgia), a biodegradable product with phages and other medications, is marketed for human application.

The study described herein is the first combining honey and phages. The chosen bacterial target was *E. coli*, one of the most frequently isolated Gram-negative pathogens from chronic wounds (Mangram et al., 1999). The effect of the combined application of both antimicrobial agents was compared with the efficacy of honey and phage alone. The phage used in this work, EC3a, was isolated from raw sewage and infected 12 of 31 multidrug resistant *E. coli* clinical isolates (Andrade, 2014).

## Materials and Methods

### Bacterial Strains and Growth Conditions

*Escherichia coli* reference strain CECT 434 was purchased from the Spanish Type Culture Collection and the clinical isolate EC3a was kindly provided by the Hospital Escala Braga in Portugal. *E. coli* EC3a was used as phage propagation strain and *E. coli* CECT 434 in all biofilm experiments. Both strains were grown at 37°C in tryptic soy broth (TSB, VWR) or in solid tryptic soy agar (TSA) medium [TSB containing 1.5% (w/v) of NZYTech agar] and for viable cell counts MacConkey Agar (Merck^®^) was used. *E. coli* K12 JM109, *Pseudomonas aeruginosa* PAO1, *Staphylococcus aureus* ATCC 19685 and a clinical isolate *Acinetobacter pittii* CEB-Ap (Oliveira et al., 2017) were included in this study to determine the minimum inhibitory concentration (MIC) of the different Portuguese honeys.

### Bacteriophage Isolation and Production

Phage vB_EcoS_CEB_EC3a, mentioned below as EC3a, was isolated from raw sewage using the clinical isolate EC3a as host (Andrade, 2014). EC3a production was carried out in its isolation host, using the plate lysis and elution method (Sambrook and Russell, 2001). Briefly, 5 μL of phage suspension were spread evenly on host bacterial lawns using a paper strip and incubated overnight at 37°C. Afterward, 3 mL of SM buffer (5.8 g.L^-1^ NaCl, 2 g.L^-1^ MgSO_4_.7H_2_O, 50 mL.L^-1^ 1 M Tris–HCl pH 7.5, VWR) were added to each plate and incubated overnight at 4°C with gentle stirring (50 rpm on a PSU-10i Orbital Shaker; BIOSAN). Subsequently, all liquid was collected, centrifuged (10 min, 9,000 × *g*, 4°C). The supernatant was collected, phages further concentrated with PEG 8000 (Thermo Fisher Scientific), purified with chloroform, filter sterilized (PES, GE Healthcare, 0.2 μm) and stored at 4°C until use. The diameter of six individual phage plaques (plaque and halo) were registered.

### Honey Samples

A total of 13 Portuguese honeys from different geographic origins, harvested during 2015–2016 were collected from regional beekeepers. All honey samples were raw and unprocessed, and were maintained in the dark at room temperature until analysis and use in antimicrobial experiments. Commercial 100% medical grade Manuka honey (Medihoney^®^, Derma Sciences) was also analyzed in this study.

### Physicochemical Characterization of Honey

The following physicochemical parameters were assessed: pH, color, and protein content (Supplementary Table S1). The pH and color were determined according to official methods recommended by the International Honey Commission and US National Honey Board (Bogdanov et al., 2002). Honeys were categorized according to the reference table of the United States Department of Agriculture (United States Department of Agriculture, 1985). The protein content of honeys was determined using the BCA Protein Assay Kit (Thermo Scientific^TM^ Pierce^TM^) following manufacturer’s instructions.

Pollen analysis and conductivity were only determined for honeys used in the antibiofilm experiments—U3 and PF2 (Supplementary Table S2), respectively.

#### Hydroxymethylfurfural Content of Honey

Hydroxymethylfurfural (HMF) content of honeys was determined using the method described by White (1979). Briefly, 0.5 g of each honey was weighed into a 50 mL flask containing 12.5 mL of water and 0.25 mL of Carrez Solution I (150 mg.mL^-1^ potassium ferrocyanide, Sigma). The solution was homogenized, 0.25 mL of Carrez solution II (300 mg.mL^-1^ zinc acetate, Biochem Chemopharma) was added and the volume adjusted with distilled water to achieve 25 mL. All solutions were filtered (PES, 0.22 μm). A volume of 2.5 mL of each honey sample was collected to two 15 mL tubes: (i) 2.5 mL of water were added to tube 1, and (ii) 2.5 mL of 0.20% (w/v) sodium bisulfite (Sigma) were added to tube 2. The solutions were mixed and turbidimetry measured at 284 nm (A_284_) and 336 nm (A_336_) (UV-3100PC VWR Spectrometer). The HMF content was calculated using the formula HMF (mg.kg^-1^ of honey) = (A_284_ - A_336_) × 149.7 × 5/[weight of sample (g)].

#### Methylglyoxal Content of Honey

MGO of honeys was detected and quantified by reverse phase- high pressure liquid chromatography (RP-HPLC) of the corresponding quinoxalines that resulted from derivatization with *o*-phenylenediamine (OPD, Amresco) (Adams et al., 2008). The RP-HPLC method was performed in a Shimadzu^®^ instrument, using a C18 column (Merck^®^). First, derivatization steps were performed, in order to obtain MGO (and DHA an OPD-derivative). For this, approximately 0.6 g of honey were dissolved in ultra-pure water [30% (w/v)] and mixed with 750 μL of 2% (w/v) OPD solution in 0.5 M phosphate buffer (pH 6.5). Samples were then incubated at room temperature in the dark for 16 h and membrane filtered (PES, 0.22 μm) before HPLC running.

The mobile phases used for HPLC were 0.075% (v/v) acetic acid (Fisher Chemical) in water (solvent A) and 80% (v/v) methanol (Biochem Chemopharma) in water (solvent B), in a 1:1 (v/v) proportion. The gradient started with 10% (v/v) solvent B for 4 min and then was elevated gradually to 100% B over a period of 31 min, and was held there for 3 min, and changed back to 10% (v/v) B in 6 min. The flow rate was 0.3 mL.min^-1^ and the separation was performed at 30°C. A volume of 20 μL of sample solution was injected and peaks were detected by measurement of UV absorbance at 312 nm. Quantification was achieved by external calibration with standard solution for MGO using a grade solvent MGO of 35–40% (v/v) (Alfa Aesar). Finally, MGO was eluted after about 21 min.

### Determination of the Minimum Inhibitory Concentration of Honey

MIC of honeys was determined using the broth microdilution method described in the guidelines of the Clinical and Laboratory Standards Institute [CLSI, Wayne, NJ, United States (M27-A2)] (Andrews and Andrews, 2001; Ferraro et al., 2003). Briefly, fresh colonies of *E. coli, P. aeruginosa, S. aureus*, and *A. pittii* were selected from TSA plates, transferred to 10 mL of TSB and incubated at 37°C, 120 rpm for 16 h. The turbidity of the bacterial culture at 620 nm was adjusted to 0.13 [approximately 3 × 10^8^ colony forming unit (CFU).mL^-1^; Synergy HT—BioTek] and diluted 30-fold in TSB. MICs were determined in a 96-well flat bottom plates (Orange Scientific) using a final volume of 100 μL. Honey concentrations ranged from 50% (w/v) to 3.125% (w/v). Plates were incubated for 20 h at 37°C and growth inhibition confirmed visually and by turbidimetry (620 nm, Synergy HT—BioTek). Five independent experiments were performed in triplicate.

### Phage DNA Extraction and Genome Sequencing

DNA of phage EC3a was extracted as described before (Melo et al., 2014). Purified phages were treated with 0.016% (v/v) L1 buffer [300 mM NaCl, 100 mM Tris–HCl (pH 7.5), 10 mM EDTA (Amresco), 0.2 mg.mL^-1^ BSA, 20 mg.mL^-1^ RNase A (Sigma), 6 mg.mL^-1^ DNase I (Sigma)] for 2 h at 37°C. The enzymes were subjected to a thermal inactivation for 30 min at 65°C. Then, 50 μg.mL^-1^ proteinase K (NZYTech), 20 mM EDTA, and 1% (w/v) SDS (Sigma) were added to digest proteins for 18 h at 56°C. This was followed by phenol:chloroform:isoamyl alcohol solution (25:24:1, v/v; Thermo Fisher Scientific) and chloroform (Thermo Fisher Scientific) extractions. DNA was precipitated with isopropanol and 3 M sodium acetate (pH 4.6) (Thermo Fisher Scientific), centrifuged (15 min, 7,600 × *g*, 4°C), and the pellet air-dried. The pellet was resuspended in nuclease-free water (GE Healthcare).

Genome sequencing was performed on an Illumina HiSeq platform (STAB VIDA). EC3a genome was mixed at equimolar ratios with a non-homologous phage, and subjected to quality controls using an Agilent Bioanalyzer. DNA library preparations were prepared using KAPA DNA Library (KAPA Biosystems) to generate 200-bp fragments with 2 × 100 bp paired-end read length configuration. After processing, reads were trimmed to remove adapters, contaminations, or low-quality sequences. Contigs were assembled with a relatively homogenous coverage with the CLC genomics Workbench version 7 (CLC Bio) using the *de novo* assembly algorithm and manual inspection. EC3a phage genome was autoannotated, using MyRAST (Aziz et al., 2008) and the presence of non-annotated CDSs, along with genes in which the initiation codon was miscalled, were checked manually using Geneious 9.1.4 (Biomatters), and potential frameshifts were checked with BLASTX (Altschul, 1997). The functions of translated open-reading frames were searched by BLASTP programs (Altschul et al., 1990) (*E* value ≤10^-5^) and HHPRED (Soding et al., 2005) server, consulted between November and December 2016. Protein parameters (isoelectric point and molecular weight) were determined using Sequence Manipulation Suite: Protein Isoelectric Point and Sequence Manipulation Suite: Protein Molecular Weight (Stothard, 2000). The presence of transmembrane domains was checked using TMHMM (Käll and Sonnhammer, 2002) and Phobius (Käll et al., 2004), and membrane proteins were annotated when both tools were in concordance. The search of tRNA encoding genes was performed using tRNAscan-SE (Schattner et al., 2005). Putative promoters were searched using PromoterHunter (Klucar et al., 2010), and putative regions were manually verified. ARNold (Naville et al., 2011) was used to predict rho-independent terminators and the energy was calculated using Mfold (Zuker, 2003). Whole-genome comparisons between EC3a and some of its closest relatives were performed using progressiveMAUVE (Darling et al., 2010) and OrthoVenn (Wang et al., 2015). Phage EC3a (KY398841) was compared with *E. coli* phages vB_EcoS_ACG-M12 (JN986845), RTP (AM156909), JK06 (DQ121662), phiJLA23 (KC333879), and T1 (AY216660).

### Assessment of Phage Viability in Honey

Phage EC3a viability was tested in PF2 and U3 honeys. Briefly, phage [2 × 10^9^ plaque forming unit (PFU).mL^-1^] was incubated with both honeys at 25% (w/v) and 50% (w/v). Honeys will be mentioned hereafter as PF2*_x_*_%_, and U3*_x_*_%_, where *x* corresponds to the honey percentages in w/v of 25 and 50, respectively. Controls were performed in sterile deionized water instead of honey. The solutions were incubated at 37°C and samples taken during 1 h until 6 h to confirm that progeny phages have then time to reach and infect a neighbor biofilm cell, and then after 12 and 24 h. Phage EC3a was quantified, at the different time-points, according to the double agar overlay technique (Adams, 1959). Briefly, 10-fold diluted phage suspension, 100 μL of host bacteria culture, and 3 mL of TSA top agar were poured onto a Petri plate containing a layer of TSA. After overnight incubation at 37°C, the PFUs were determined. Three independent experiments were performed.

### Biofilm Formation and Treatment

For biofilm formation, the turbidimetry of a 16 h *E. coli* inoculum grown in TSB was adjusted to 0.13, and diluted 10-fold in TSB. After, 200 μL were added to wells of a 96-well plate and plates incubated for 24 h or 48 h, at 37°C and 120 rpm (Orbital Shaker ES-20/60; BIOSAN). In 48-h-old biofilms, at 24 h, 150 μL medium were replaced with fresh TSB.

Three different biofilm treatments were evaluated: phage, honey, and the combination of both agents. Phage treatments were performed with a multiplicity of infection (MOI) of 10, honey challenging was done with 25% (w/v) and 50% (w/v) concentrations, and the combinatorial effect of phage–honey was accomplished using the concentrations used in the single-agent experiments. Biofilms formed on 96-well plates as described above, were washed twice with saline [0.9% (w/v) NaCl, VWR] to remove all non-adhered cells. After, 200 μL of phage or honey or combination of both was added to each well and the plates incubated at 37°C, 120 rpm (Orbital Shaker ES-20/60; BIOSAN). The control biofilms of each treatment were performed in 100 μL 2× TSB, and 100 μL SM buffer. Samples were taken at 0, 6, 12, and 24 h for viable cell quantification. Three independent experiments were performed.

### Viable Biofilm Cell Quantification

Viable cells in biofilms were quantified according to a previously described procedure (Pires et al., 2011), with some modifications. Briefly, honey and/or phage treated biofilm and also non-treated controls were washed twice with saline, saline (200 μL) was then added to each well, and with the aid of a pipette tip all biomass detached from the bottom and walls. Serial dilutions were performed in saline containing 1 mM ferrous ammonium sulfate (Applichem Panreac) to assure that all non-infecting phages were destroyed (Park et al., 2003). Samples (10 μL) were plated on MacConkey Agar using the microdrop technique (Naghili et al., 2013), plates incubated 16 h at 37°C, and CFUs determined.

### Susceptibility of Surviving Biofilm Cells to Phage

Distinct colonies (19–21 colonies) remaining after phage and combined phage–honey treatments were tested for their susceptibility to phage EC3a using the method of streaking a line of cells through a perpendicularly streaked line of phage solution (Moons et al., 2013).

### Scanning Electron Microscopy Analysis

For scanning electron microscopy (SEM) visualization, biofilms were formed on polystyrene coupons (Nunc^TM^ Thermanox^TM^, Thermo Scientific^TM^) placed on 24-well plates. Biofilm formation and treatment was done as described above with volumes of bacterial inoculum, honey and/or phage and solutions used in the control assays adapted for a volume of 1 mL instead of 200 μL. Biofilms were washed twice with saline and fixed with 2.5% (v/v) glutaraldehyde (Thermo Fisher Scientific). Coupons were left at 4°C for 1 h and sample dehydration was carried out in ethanol series [30, 50, 70, 80, 90% (v/v), and absolute] (Fisher Chemical). Biofilms were coated with gold and analyzed by NanoSEM [FEI Nova 200 (FEG/SEM); EDAX—Pegasus X4M (EDS/EBSD)]. The lengths and diameters of 6–10 untreated and treated *E. coli* cells were measured.

### Transmission Electron Microscopy Analysis

Phage EC3a particles, before and after 6 h contact with honeys PF2 and U3 at 25% (w/v) concentration, were sedimented by centrifugation (25,000 × *g*, 60 min, 4°C) and washed twice in tap water by repeating the centrifugation step. Subsequently, the suspension was deposited on copper grids with carbon-coated Formvar films, stained with 2% (w/v) uranyl acetate (pH 4.0) (Agar Scientific), and examined using a Jeol JEM 1400 (Tokyo, Japan) transmission electron microscope (TEM).

*Escherichia coli* cells challenged with phage for 2 h, honey and the combination of both for 12 h, were also visualized by TEM along with the respective control samples. In brief, samples were fixed with 2.5% (v/v) glutaraldehyde (Electron Microscopy Sciences, Hatfield, United States) and 2% (v/v) paraformaldehyde (Merck, Darmstadt, Germany) in phosphate buffer 0.1 M with 0.5 mM MgCl_2_ (pH 6.5), dehydrated and embedded in Epon resin (TAAB, Berks, England). Ultrathin sections (40–60 nm thickness) were prepared on a RMC Ultramicrotome (PowerTome, United States) using diamond knives (DDK, Wilmington, DE, United States). The sections were mounted on 200 mesh copper or nickel grids, stained with 2% (w/v) uranyl acetate and 3% (w/v) lead citrate for 5 min each, and examined under by TEM (Jeol JEM 1400, Tokyo, Japan). Images were digitally recorded using a CCD digital camera Orious 1100W, Tokyo, Japan.

### Zeta Potential

Zeta potential of *E. coli* 24-h-old biofilm cells after 6, 12, and 24 h treatment with phage, honey, and the combination of both was determined by dynamic light scattering with a Malvern Zetasizer, NANO ZS (Malvern Instruments Limited) and values were calculated using the Smoluchowski equation (Hunter, 1981). Briefly, biofilms were formed and treated as described above, and after each treatment, wells were washed thrice with saline, and surfaces scratched to detach biofilms. Samples (1 mL) were collected into a 2 mL tube, homogenized and diluted 10-fold in Milli-Q^TM^ water prior to each analysis. Each data value is an average of three independent zeta potential measurements.

### Flow Cytometry Analysis

Cell viability before and after single and mixed treatments was assessed by flow cytometry as previously optimized (Cerca et al., 2011) with some modifications. In brief, biofilms were washed as described above, and resuspended in 200 μL. Then, 20 μL of suspension was added to 180 μL of a solution containing 250 nM of SYTO^®^ BC Green Fluorescent Nucleic Acid Stain (Thermo Fisher Scientific) and 20 μg.mL^-1^ of propidium iodide (PI) (Thermo Fisher Scientific). The fluorescence of bacteria was measured using an EC800 (SONY, San Jose, CA, United States) flow cytometer. SYTO^®^ BC was detected on the FL1 channel and PI on the FL4 channel. For all detected parameters, amplification was carried out using logarithmic scales. Data were acquired and analyzed using Sony EC800 Flow Cytometry Analyzer software. Two independent experiments were performed in duplicate. Live cells counts were determined subtracting the number of events that were SYTO BC positive from the PI^+^ events.

### Statistical Analysis

Statistical analysis of the results was performed using GraphPad Prism 6. Mean and standard deviations (SD) were determined for the independent experiments and the results were presented as mean ± SD. Results were compared using two-way ANOVA, with Turkey’s multiple comparison statistical test. Differences were considered statistically different if *p* ≤ 0.05 (95% confidence interval).

## Results

### Honey Samples

Several honey samples collected from regional beekeepers were characterized (Supplementary Table S1). Based on two main characteristics—MGO content, a molecule reported as the major antibacterial agent in honeys (Kilty et al., 2011), and MIC, two honeys were selected for further characterization and antimicrobial evaluation in *E. coli* biofilms. The honey with lowest MIC [12.5% (w/v)] toward *E. coli* 434 was PF2 with an MGO content of 316.6 mg.kg^-1^. The MIC of honeys tested against another *E. coli* and toward *S. aureus, P. aeruginosa*, and *A. pittii* revealed that 25% (w/v) was the predominant MIC value of honeys but could decrease to as low as 3.125% (w/v) as observed with *S. aureus* with honey E1. The honey with highest MGO content was U3 with 2092.4 mg.kg^-1^ and a MIC of 25% (w/v). PF2 is a polyflora honey having two main floral sources [*Castanea sativa* (56%) and *Eucalyptus* spp. (26%)] (Supplementary Table S2) and is a light amber honey with a total protein content of 16.6 mg.g^-1^, HMF 47.9 mg.kg^-1^, pH of 4.13, and a conductivity of 753 μS.cm^-1^. U3 honey is also polyflora (30% *Erica* spp., 21% *Rubus* spp./*Eriobotrya* spp., and 18% *C. sativa*) and is dark amber, with a total protein content of 81.7 mg.g^-1^, HMF 204.6 mg.kg^-1^, pH 4.04, and a conductivity of 622 μS.cm^-1^.

### Morphology and Genome of Phage EC3a

**FIGURE 1:**
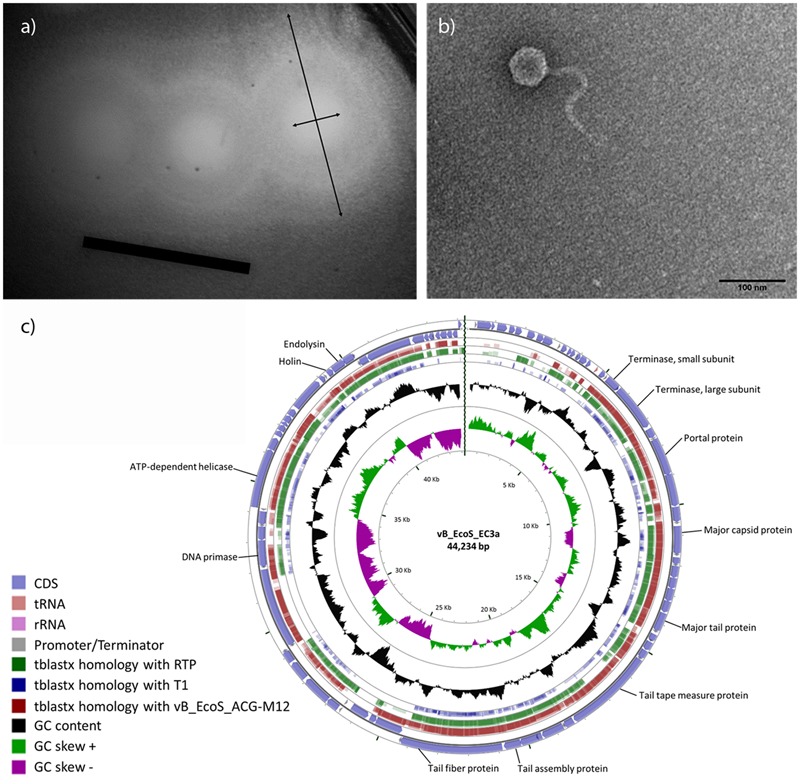
Characteristics of EC3a. **(a)** Plaque morphology (black arrows indicate diameter of EC3a plaque and diameter of EC3a plaque and the surrounding halo. Scale bar 1 cm), **(b)** virion particle, and **(c)** circular view of phage vB_EcoS_CEB-EC3a and TBLASTX comparison with the two closest *E. coli* phage homologs and T1. The outer ring represents EC3a CDSs. The other three outer rings represent TBLASTX homologies with phages M12, RTP, and T1, respectively. The GC content appears in the black ring, and the inner rings are the GC skew^+^ (green) and the GC skew^-^ (pink). Some important EC3a genes are indicated.

EC3a forms clear plaques (Ø_plaque_ = 2.63 ± 0.35 mm) surrounded by a halo (Ø_plaque+halo_ = 11.55 ± 1.61 mm) (**Figure 1a**). EC3a has an icosahedral head with a diameter of 57 nm tapered with a non-contractile tail of 192 nm × 11 nm with conspicuous striations, resembling members of the T1-like phages of the *Siphoviridae* family (**Figure 1b**).

EC3a does not encode known genes associated with lysogeny or toxin proteins, suggesting that it is potentially safe for therapy applications. The genome consists of a linear double-stranded DNA with 44,234 bp with a G+C% content of 44.2% (**Figure 1c**). EC3a encodes 70 open reading frames (ORFs), tightly packed occupying 91% of its genome. Twenty-five of the predicted ORFs (36%) have an assigned function and four are unique (Supplementary Table S3). Furthermore, one tRNA gene (tRNA-Arg), 10 promotors, and 16 rho-independent terminators were predicted. BLASTN search revealed homology of EC3a with *E. coli* siphoviruses vB_EcoS_ACG-M12 (77% coverage; 93% identity) and RTP (68% coverage; 89% identity). Furthermore, OrthoVenn showed that approximately 83% of EC3a genes are orthologous to vB_EcoS_ACG-M12 and RTP, 70% to phage phiJLA23, 61% to JK06, and 54% to phage T1. This suggests that EC3a is phylogenetically related with vB_EcoS_ACG-M12 and RTP and these three phages reunite the conditions for the formation of a new genus. All EC3a ORFs were analyzed with HHpred to identify a putative depolymerase which could explain the halo formation (**Figure 1a**). ORF 35, a minor tail protein (Supplementary Table S3) was found to present a cysteine peptidase domain [NLPC_P60 (PF00877)] that is a putative tail-associated endolysin domain.

### Viability of Phage EC3a upon Contact with Honey

**FIGURE 2:**
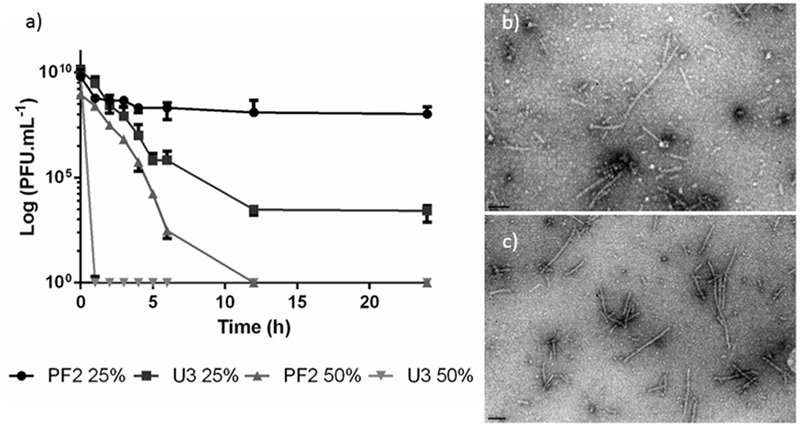
Phage EC3a viability. **(a)** PFU counts after EC3a exposure to PF2 and U3 honeys at 25% (w/v) and 50% (w/v) concentrations, TEM micrographs of EC3a phage tails after 6 h of contact with honeys with **(b)** U3_25%_ and **(c)** PF2_25%_. Scale bar in TEM micrographs is 100 nm.

Before antimicrobial experiments, the viability of phage was inspected in PF2 and U3 honeys. EC3a was exposed to both honeys at two concentrations—25% (w/v) and 50% (w/v) (**Figure 2a**). In PF2_25%_, there was only minor loss in viability and EC3a concentration remained fairly stable along 24 h of incubation. PF2_50%_ presented 3 × 10^2^ PFU.mL^-1^ at 6 h, however, at 12 h no viable phages remained.

The viability of EC3a in U3_25%_ progressively decreased with time until 12 h and by the end of 24 h incubation 2.6 × 10^3^ PFU.mL^-1^ were still present in the samples. On the other hand, U3_50%_ completely inactivated EC3a within 1 h upon contact.

TEM imaging confirm that both honey presents antiviral activity toward the phage (**Figures 2b,c**).

### Antibiofilm Effect of Phage, Honey, and Phage–Honey Combination on 24-h-Old Biofilms

**FIGURE 3:**
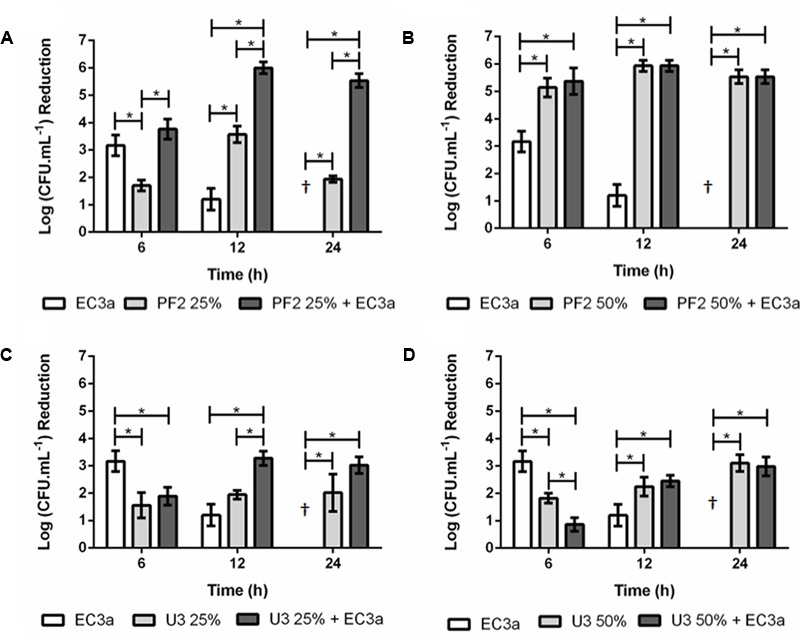
Antibiofilm effect of phage EC3a, honey, and of the phage–honey combination on 24-h-old *E. coli* biofilms. **(A)** PF2_25%_, **(B)** PF2_50%_, **(C)** U3_25%_, **(D)** U3_50%_. ^†^Values present no reductions compared to control samples. ^∗^Indicates that the difference is statistically significant at the *p*-value < 0.05 level.

The effect of phage, honeys (PF2 and U3), and the phage–honey combinations was evaluated in 24 h *E. coli* biofilms (**Figure 3**).

#### Single Agent Approach

Maximum antimicrobial activity in 24-h-old biofilms challenged with EC3a phage occurred after 6 h treatment resulting in viable cell reductions of 3.2 log. The increase of treatment to 12 h showed less evident efficacy of EC3a (*p* < 0.05) and at 24 h no apparent antibacterial of EC3a effect was perceived. PF2_25%_ and PF2_50%_ resulted in the highest viable cell reductions at 12 h which were approximately 3.6 and 5.9 log, respectively (**Figures 3C,D**). The use of U3 honey, at both tested concentrations was not as efficient as PF2 honey and phage EC3a (*p* < 0.05) in decreasing biofilm cells at 6 h of treatment. Furthermore, at 6 h of treatment U3 was less efficient than the phage itself. This tendency was, however, inverted at 12 and 24 h. Overall, for a short (6 h) treatment of *E. coli* biofilms the best selections using a single agent approach were obtained using phage EC3a (3.2 log viable cell reduction) or PF2_50_ (5.1 log viable cell reduction). Biofilm treatment during 12 h is more efficient using PF2_25%_ or PF2_50%_ (3.6 and 5.9 log viable cell reductions, respectively) and 24 h treatments were best with PF2_50%_ and U3_50%_ (5.5 and 3.1 log viable cell reductions with PF2 and U3, respectively).

#### Phage–Honey Approach

The sum of viable cell reduction caused individually by phage and PF2_25%_ was 4.8 logs at 12 h and 1.9 logs at 24 h. The combined phage–PF2_25%_ strategy produced a synergistic effect that resulted in a 5.9-log reduction at 12 h and 5.5-log reduction at 24 h, respectively. Even though the reduction of viable cells at 6 h was greater in the combined treatment, no additive or synergic effect was detected. The viable cell reductions using PF2_50%_ and phage–PF2_50%_ were equivalent (*p* > 0.05) and statistically higher than the action of phage itself (*p* < 0.05). At 6 h, the antimicrobial action of phage–U3_25%_ was significantly lower (*p* < 0.05) than the effect achieved with phage, and similar (*p* > 0.05) to U3_25%_ alone. However, after 12 h of treatment, phage–U3_25%_ displayed an additive antimicrobial effect (*p* < 0.05) compared to the treatment with each components individually (**Figure 3C**). No significant differences were observed between U3_25%_ and phage–U3_25%_ at 24 h post-treatment (*p* > 0.05) showing that honey alone or combined with phage induced a similar response.

In general, these results point to a better biofilm control using the phage–PF2 honey combination rather than the phage–U3 honey combination.

### Antibiofilm Effect of Phage, Honey, and Phage–Honey Combination on 48-h-Old Biofilms

**FIGURE 4:**
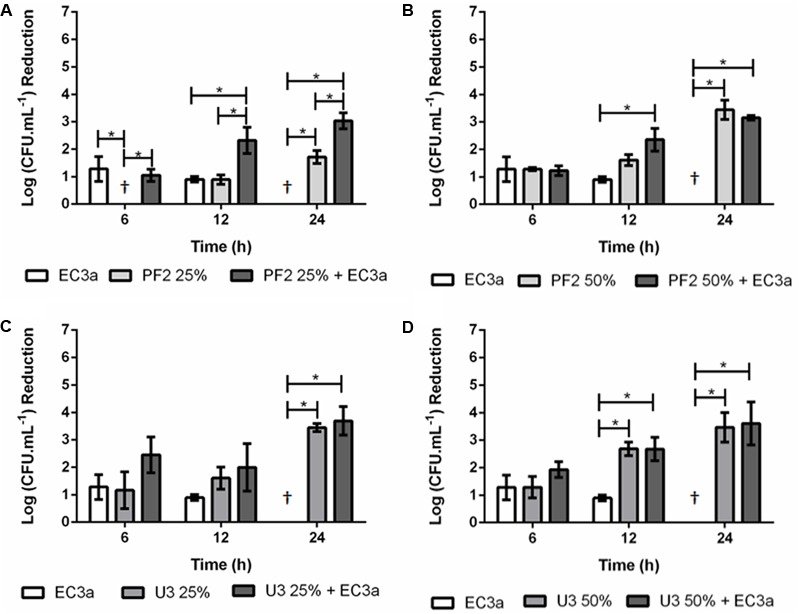
Antibiofilm effect of phage EC3a, honey, and of the phage–honey combination on 48-h-old *E. coli* biofilms. **(A)** PF2_25%_, **(B)** PF2_50%_, **(C)** U3_25%_, **(D)** U3_50%_. ^†^Values present no reductions compared to control samples. ^∗^Indicates that the difference is statistically significant at the *p*-value < 0.05 level.

The effect of phage and the two honeys concentrations individually and combined was also evaluated in 48 h *E. coli* biofilms (**Figure 4**).

#### Single Agent Approach

Phage EC3a had minor and statistically similar (*p* > 0.05) effect in 48-h-old biofilms treated during 6 and 12 h, and no influence in reducing viable cells after 24 h. The effect of PF2 honey, at both tested concentrations (**Figures 4A,B**), was less efficient than the treatment effect obtained for 24-h-old biofilms (**Figure 3**). PF2_25%_ had no effect after 6 h, and only minor decrease of viable cell counts at 12 and 24 h (0.9 and 1.7 log, in average), respectively. Treatment with PF2_50%_ (**Figure 4B**) exhibited slight antimicrobial effect at 6 and 12 h (1.3 and 1.6 log reduction of viable cells), increasing significantly (*p* < 0.05) to 3.4 log viable cell reduction after 24 h. The effect of U3 honey in 48-h-old biofilms resulted in similar (*p* > 0.05) viable cell reductions with both concentrations (3.4 and 3.5 log with U3_25%_ and U3_50%_, respectively) (**Figures 4C,D**).

#### Phage–Honey Approach

Phage–PF2_25%_ effect was as follows: at 6 h the effect observed was mostly due to phage infection since the viable cell counts of the combinatorial treatment were identical to the phage results alone; at 12 h the phage–honey combination acted synergistically [sum of individual treatment (1.8 log) < result of combined treatment (2.3 log)] and synergism maintained until the last time-point of treatment [sum of individual treatment (1.7 log) < result of combined treatment (3.0 log)]. Phage–PF2_50%_ reduced as many viable cell counts at 6 h as each agent individually. The increase of treatment to 12 h favored the combinatorial approach (2.5 log viable cell reduction), that produced an additive effect, in detriment of each individual approach (0.9 and 1.6 log viable cell reductions with EC3a and PF2_50%_, respectively). The results recorded at 24 h revealed no differences between the use of honey combined with phage or honey alone (*p* > 0.05). A comparison of the effect of the phage–PF2 honey in 24- and 48-h-old biofilms reveals much higher efficacy of this honey against younger (>5.5 log reduction of viable cells) than older (∼3 log reduction of viable cells) biofilms. Phage–U3_25%_ resulted also in an additive effect at 6 h, however, this combination after 12 and 24 h of treatment resulted in cell reductions similar to the ones registered for honey alone. Since there was no action of the EC3a at 24 h, the result observed was only due to the antimicrobial effect of honey. The same trend was observed with phage combined with U3_50%_ after 12 and 24 h of treatment.

### Susceptibility of Surviving Cells to Phage EC3a

The susceptibility of surviving colonies from 24-h-old biofilms challenged with phage EC3a and the combined phage–honey treatment to EC3a was tested. With the exception of 1 colony obtained after combined treatment of phage–U3_50%_, all other surviving cells from phage–honey treatments were susceptible to EC3a. On the other hand, 28.6% (6 out of 21 colonies assessed) of colonies isolated after phage single treatment were insensitive to EC3a (data not shown).

#### Insights of the Different Treatment Effects on *E. coli*

##### Scanning electron microscopy analysis

**FIGURE 5:**
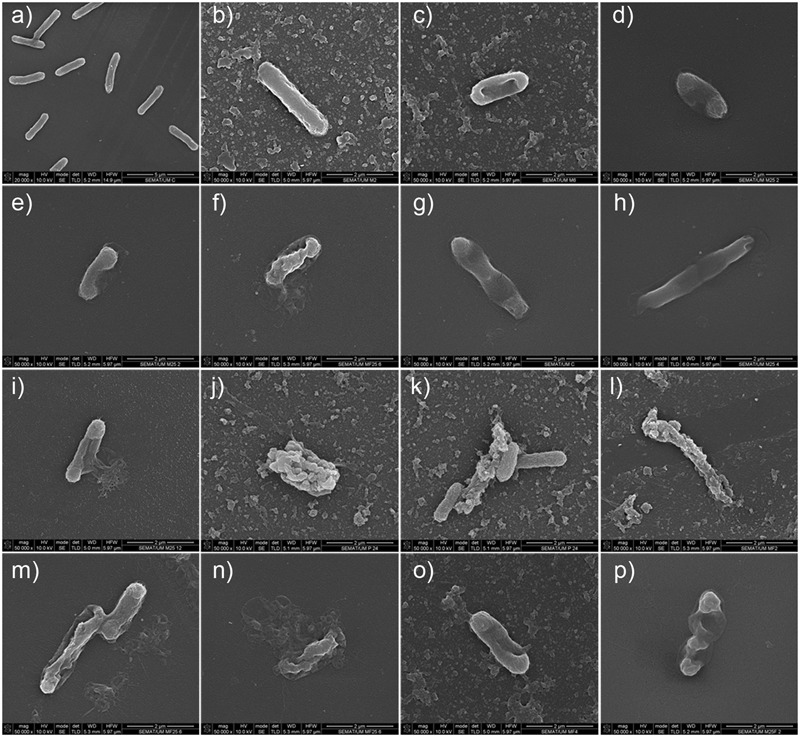
SEM micrographs showing the effect of honey, phage, and honey–phage combination treatments in *E. coli* cells. **(a)** Control *E. coli*, **(b–i)** honey treatment, **(j,k)** phage treatment, **(l–p)** phage–honey treatment. The honey used in these experiments was PF2.

Cell morphology was assessed by SEM before and after honey, phage, and combined honey–phage treatments to assess possible changes. *E. coli* has a typical rod-shape form (length of 2.04 ± 0.26 μm and diameter of 0.54 ± 0.02 μm) (**Figure 5a**). All treated cells retained their rod-shape morphology; however, considerable phenomena associated to the exposure to honey was detected. For instance, honey-mediated treatment caused: (i) minor perturbations on the bacterial envelope unseen in control *E. coli* (**Figure 5b**), (ii) shrinkage of cells with small and pronounced collapsing of the bacterial envelope in the septal and apical regions (**Figures 5c–f**), (iii) membrane ruffling and/or possible membrane detachment (**Figures 5d,e**); (iv) membrane disruption and leakage of the cytoplasmic content and debris around the disintegrated cells (**Figures 5f,j**). Structural alterations of the outer membrane such as disruption caused cytoplasmic leakage from the apical and septal regions, and these observations happened in the majority of cells imaged after 12 and 24 h. Shrunk *E. coli* cells were shorter in length (1.79 ± 0.20 μm) and had either wider (0.77 ± 0.09 μm) or thinner (0.45 ± 0.07 μm) diameter in the mid-region area. Besides shrinkage, honey disabled cell division (**Figures 5g,h**). Cells exposed to phage showed formation of vesicle-like structures, and in general higher amounts of cell debris on the polystyrene surfaces (**Figures 5i,j**). The combined phage–honey treatment exhibited a mixture of cell damage phenomena similar to the observed in the individual treatments (**Figures 5k–p**). SEM observations did not show simultaneous effect of both agents in an individual cell. Changes of the *E. coli* morphology with honey correlate with viability experiments (**Figures 3, 4**).

##### Transmission electron microscopy analysis

**FIGURE 6:**
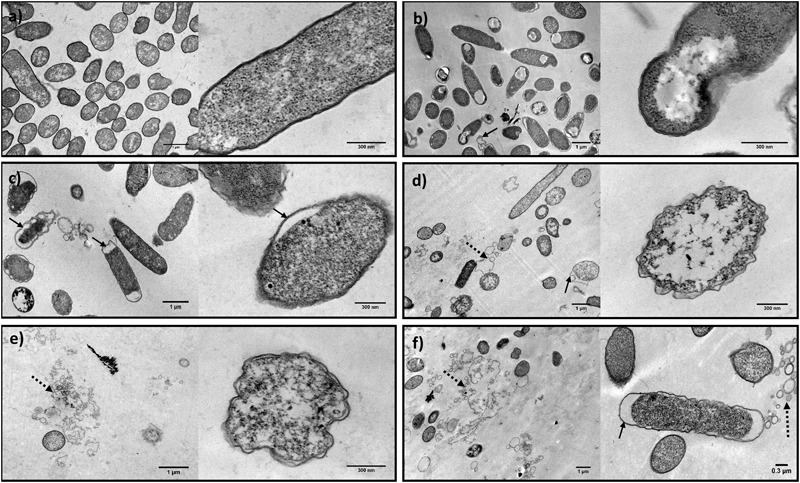
TEM micrographs showing the effect of honey, phage, and honey–phage combination treatments in *E. coli* cells. **(a)** Control *E. coli*, **(b)** PF2_25%_, **(c)** U3_25%_, **(d)** EC3a phage at a MOI 10, **(e)** phage–PF2_25%_ at a MOI 10, **(f)** phage–U3_25%_ at a MOI 10 (arrows indicate membrane detachment and dashed arrows point cell debris).

Untreated *E. coli* cells showed regular morphology, with intact cell envelopes (**Figure 6a**). In the treated samples—PF2_25%_; U3_25%_; phage EC3a; phage–PF2_25%_; phage–U3_25%_—it was possible to observe morphological and integrity changes in the surface layers and alterations in the cytoplasm density (**Figures 6b–f**).

Honey and honey–phage effect were observed after 12 h treatment, the time-point which provided synergistic effect. Honey treatment apparently disclosed integral outer membranes, however, with obvious damages in the inner cell. While U3 revealed a detachment of the outer membrane more notorious in the apical sites, PF2, besides the same detachment phenomenon, was able to induce cytoplasm-clearing sites in one zone of the cell (**Figures 6b,c**). In both cases, the condensation of the cytoplasmic material was noticed.

Phage EC3a effect on *E. coli* cells was analyzed by TEM after 2 h of treatment, taking into account the moment that phage start to cause cell damage, but not total destruction that would only result in images showing cell debris. Treatment with EC3a, revealed evident irregularities in the cell wall shape. Besides, it was possible to observe some outer membrane detachment, a less dense cytoplasm and even cellular debris (**Figure 6d**).

The images obtained with the combination of phage and both honeys revealed, besides the cell structure alterations reported to each one separately, an increase on the amount of cell debris (**Figures 6e,f**).

### Zeta Potential

Measurements of zeta potential were carried out in 24-h-old biofilm cells after 6, 12, and 24 h (Supplementary Figure S1). The contribution of phage and honey to the background conductivity of *E. coli* biofilm cells was found to be negligible with a variation of no more than 0.3 mS.cm^-1^ (data not shown). At 6 h, control and phage-treated samples had more negative and significantly similar (*p* > 0.05) zeta potential values. Although all other samples resulted in an increase in zeta potential of approximately 3.9–8.8 mV (to less negative values), a statistical difference (*p* < 0.05) was only observed for phage–honey [50% (w/v)] treated biofilms compared to control. The two concentrations of honey tested [25% (w/v) and 50% (w/v)] imparted a similar surface charge neutralizing effect. Samples analyzed after 12 h of treatment yielded slight increase of zeta potential (less negative values), however, only phage-treated biofilm cells were statistically different (*p* < 0.05) compared to 6 h of treatment. After 24 h of treatment, statistical differences (*p* < 0.05) in comparison to the previous time-points were only obtained for phage and combined phage–honey [50% (w/v)] treated samples. Throughout the experiment, the sample pH values varied dimly between 5 and 6, with slightly more acidic pH obtained for honey concentrations of 50% (w/v) due to the low pH of honey itself. All zeta potential measurements were performed immediately following sample dilution (1:10) in Milli-Q^TM^ water and thus potentially confounding influences arising from variations in pH were minimized.

### Treated and Untreated *E. coli* Biofilm Analysis by Flow Cytometry

**FIGURE 7:**
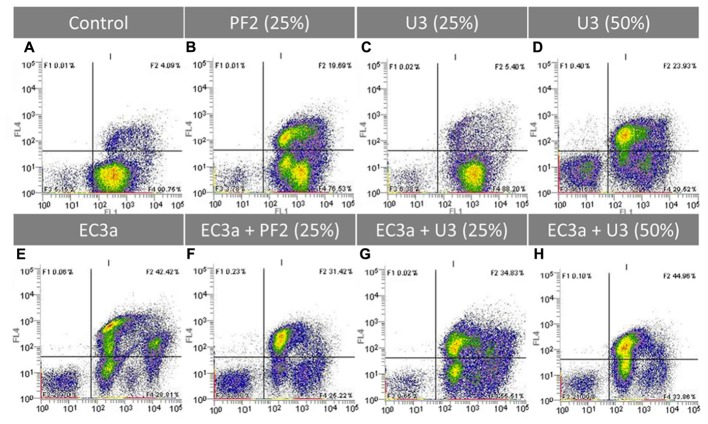
Flow cytometric analysis after 12 h application of single and combined treatments to 24-h-old *E. coli* biofilms. Representative dot plot FL1 (*x*-axis) vs FL4 channel (*y*-axis) showing *E. coli* cells stained with SYTO BC (250 nM) and PI (20 μg.mL^-1^). **(A)** *E. coli* control, **(B)** PF2_25%_, **(C)** U3_25%_, **(D)** U3_50%_, **(E)** EC3a phage at a MOI 10, **(F)** phage–PF2_25%_ at a MOI 10, **(G)** phage–U3_25%_ at a MOI 10, **(H)** phage–U3_50%_ at a MOI 10. Results are a representative example of two independent experiments.

Flow cytometry using LIVE/DEAD staining was performed to assess the effect of all treatments on the viability of 24-h-old biofilm cells (**Figure 7** and Supplementary Table S4). *E. coli* control biofilms (**Figure 7A**) presented a great number of viable cells as evidenced by the SYTO^®^ BC (SYTO) uptake (SYTO^+^/PI^-^) and also a small fraction of compromised/injured cells (SYTO^+^/PI^+^). After 12 h of treatment, PF2_25%_ and U3_50%_ resulted in a substantially higher number of compromised cells compared to the control (**Figures 7B,D**). On the other hand, U3_25%_ had a similar amount of compromised cells than the control biofilm (**Figures 7A,C**). Unexpectedly, all honeys, regardless of the concentration used, caused an increase of the SYTO mean fluorescent intensity from approximately 754 to 2.552, 2.525, and 1.666 for PF2_25%_, U3_25%_, and U3_50%_, respectively (Supplementary Table S4). Phage alone clearly reduced the viable cell population, caused an increase of damaged cells, and a higher amount of cell debris (**Figure 7E**) compared to control biofilm (**Figure 7A**) resulting in a decrease of approximately 1.5 log cells.mL^-1^. The amount of cell debris (SYTO^-^/PI^-^) was greater using phage than both honeys at 25% (w/v) (compare **Figures 7B,C,E**) but not at all comparable to the amount of debris after treatment with U3_50%_.

The comparison of single agent treatments (**Figures 7B–E**) with the combined treatments (**Figures 7F–H**) gives us an indication of the population shifts as a result of the strategy applied. The cytograms of the treatment with phage–PF2_25%_ show a synergic effect (1.82 > 0.15 + 1.52) (Supplementary Table S4). This observation is the result of the following findings: (i) the core population was similar to the one obtained with honey and the phage was able to infect the live cells that had the highest uptakes of SYTO; (ii) an increase of cell debris compared to phage and honey single treatments; (iii) less live cells compared to single treatments.

The phage–U3_25%_ combination shaped massively the biofilm driving into a diversified population in the live and compromised quadrants (compare **Figures 7C,E,G**). It is perceptible that both agents have a role in the progression of the biofilm treatment evidenced by: (i) the population with the lowest SYTO uptake, visible after phage single treatment, was present in the combined strategy; (ii) the live population present in U3_25%_ was targeted by EC3a reducing the total counts of this population.

The use of phage–U3_50%_ resulted in a lower amount of cell debris than each single treatments, and similarly to U3_25%_, phage targeted preferentially cells with higher SYTO uptake (compare **Figures 7D,E,H**).

## Discussion

*Escherichia coli* is frequent in the complex microenvironment of chronic wound bed along with *S. aureus* and *P. aeruginosa* (Moet et al., 2007; Petkovšek et al., 2009). Besides its relevance in chronic wounds, *E. coli* is also the leading cause of urinary tract infections, and one of the most common pathogens causing blood stream infections, among other infections (Kerrn et al., 2002; Kaper et al., 2004; Marrs et al., 2005; Rodriguez-Bano et al., 2010; Martelius et al., 2015). Furthermore, the increasing rates of antimicrobial resistance among *E. coli* are a growing concern in both developed and developing countries (Von Baum and Marre, 2005; Da Silva et al., 2012; Tadesse et al., 2012). Antibiotic alternatives are therefore desired.

This study evaluated the individual and combined effect of two antimicrobial agents—honey and phages. To our knowledge, this is the first study assessing the impact of honey combined with phages on the tested organism at both biofilm and cell structural levels.

Honey was chosen based on the knowledge that at least two complementary mechanisms are responsible to combat infections: (i) the direct biocidal activity, due to the presence of multiple factors that can damage susceptible organisms (high sugar content, low pH, the generation of hydrogen peroxide, bee defensin-1, various phenolic compounds and complex carbohydrates, and the MGO) that combined enable prevention and elimination of established biofilms and enhance wound healing (Lu et al., 2014); and (ii) the anti-virulence activity, through downregulation of expression of genes associated with virulence factor production, stress tolerance, and/or multicellular behaviors of the target organism (such as biofilm formation and quorum sensing) (Wang et al., 2012), that is thought to weaken bacterial coordination, decrease their survival abilities, and interfere with their virulence mechanisms (Wasfi et al., 2016). Portuguese honeys, 13 in total, were physicochemically characterized and their MIC assessed. All honeys had MICs of 12.5% (w/v) or 25% (w/v) and thus one honey presenting each MIC was selected. The honey selected for MICs of 12.5% (w/v) and 25% (w/v) were PF2 and U3, respectively. Since MGO has been identified as the dominant active antibacterial component of Manuka honey (Mavric et al., 2008; Adams et al., 2009), this criteria was used for the selection of the two honeys. However, other physical properties, known for their antimicrobial role could have also have been adopted, i.e., pH and HMF content, among others. Although MGO plays an important role in Manuka honey, a previous study using non-manuka honeys against *Clostridium perfringens* suggests that other unknown factors, rather than MGO, have a major role in the antimicrobial effect observed (Oinaala et al., 2015).

Phages were selected due to their ability to target antibiotic-resistant bacteria and bacterial biofilms, and due to the potential depolymerases that they carry that allow their entry into the inner layers of the biofilms by degrading components of the exopolymeric matrix (Bartell et al., 1966; Hughes et al., 1998; Sillankorva et al., 2004; Pires et al., 2016). The phage tested herein, phage EC3a, belongs to the *Siphoviridae* family, possess a large halo (**Figure 1**), indicative of depolymerase activity (Pires et al., 2016), and does not carry genes associated with lysogeny or toxin proteins (Supplementary Table S3). Comparative analysis of the genome of EC3a suggests that EC3a along with its phylogenetically related vB_EcoS_ACG-M12 and RTP phages could form a new genus. Typical putative lyase domain was not found in EC3a, nevertheless, there is an active peptidase domain in EC3a’s minor tail protein (ORF 35) that might be responsible for the halo formation. Exopolysaccharide depolymerase activity associated with tail fibers or with tail-spikes has been described for *Acinetobacter baumannii* phage φAB6, *Erwinia amylovora* phage L1, *Pseudomonas putida* phage φ15, among others (reviewed in Pires et al., 2016).

Maximum reduction of viable cells caused by phage EC3a was observed at 6 h; however, from this point forward the antibiofilm efficacy declined until no effect was perceived at 24 h of treatment (**Figures 3, 4**). This is in agreement with several works which argument that short phage treatments are effective nevertheless extended treatments cause regrowth of bacteria in biofilms (Cornelissen et al., 2011; Pires et al., 2011; Chibeu et al., 2012). Regrowth can be a consequence of dynamic adaptation or bacterial tactics to avoid, circumvent or subvert phage infection (Labrie et al., 2010). Regrowth of *E. coli* beyond 6 h of EC3a phage treatment can be due to a fraction of the population which became insensitive (28.6%) to the phage. These insensitive mutants continue to thrive and secrete EPS that can mask phage host receptors preventing adsorption of phages to the cells (Labrie et al., 2010).

Both Portuguese honeys were tested at 25% (w/v) and 50% (w/v) concentrations. These concentrations (2 × MIC and 4 × MIC for PF2 and 1 × MIC and 2 × MIC for U3), were able to reduce *E. coli* viable cells from 24-h-old biofilms already in 6 h, however, the higher honey concentrations were far more effective. This study shows that the active substances in honey were able to diffuse through the matrix of established *E. coli* biofilms reaching and causing damage to the bacterial cells. The effectiveness of PF2 honey was, however, highly influenced by biofilm age, resulting in lower viable cell reductions after treatment of mature biofilms (48-h-old). On the other hand, U3’s effect was not at all influenced by the age of the biofilms treated. A possible explanation for this fact might be related to the high MGO content present in this honey since this molecule holds strong antimicrobial and antibiofilm activity even in mature biofilms (Paramasivan et al., 2014). Comparing the results using 2 × MIC (PF_25%_ and U3_50%_), it is clear that PF2 is significantly more effective in reducing viable cell counts. It has been previously reported that chestnut (*C. sativa*) honeys presented high antibacterial activity against *E. coli* while *Erica* honeys had no antimicrobial activity against two reference strains *E. coli* (ATCC 25922) and *Salmonella* serovar Infantis (ATCC 1523), respectively (Coniglio et al., 2013). Although both honeys tested in our work were polyfloral, PF2 presented a predominant pollen of *C. sativa* (56%) while U3 had *Erica* spp. (30%) as the most dominant secondary pollen (Supplementary Table S2). Therefore, this higher antibacterial effectiveness of PF2 compared to U3 is most probably due to the botanical source of the honeys.

Previous works with Manuka honey have reported biomass reductions from established biofilms and impaired cell adhesion of *Streptococcus pyogenes, S. aureus*, and *P. aeruginosa* when 17% up to 40% (w/v) concentrations were used to supplement the media were biofilms were formed (Maddocks et al., 2012, 2013; Cooper et al., 2014). Furthermore, in these works, honey’s effect in cell viability in developing and established biofilms was evaluated by microscopy (LIVE/DEAD staining), where honey was shown to increase the number of dead cells (Maddocks et al., 2012, 2013; Cooper et al., 2014). Manuka did not eradicate these pathogens, and therefore the biomass reductions were suggested to be due to a combined effect of growth inhibition and cell death. In our work, flow cytometry experiments did not show any increase of cell death (SYTO^-^/PI^+^ quadrants in **Figures 7B–D**), however, honey was greatly responsible for an increase of cells with compromised membrane as visible in the SYTO^+^/PI^+^ quadrants. Furthermore, both tested honeys caused an increase of the uptake of SYTO which has been described as a consequence of the process of cells becoming permeabilized (Berney et al., 2007). During the permeabilization event, intermediate states in *E. coli* biofilm cells are occurring which result in different intracellular concentrations of SYTO as a result of the degree of destruction distressed on the bacterial membrane. Even though the percentage of live cells after exposure to honey was still high, these living cells were not able to grow once plated on agar plates (Supplementary Table S4). In terms of flow cytometry cell counts, the antibiofilm treatments caused reductions between 0.15 up to 1.82 log cells.mL^-1^ while the culturable cell counts after treatment varied in the range of 1.21 to more than 5.85 CFU.mL^-1^. For instance, at 12 h of treatment, PF2_25%_ honey resulted in culturable cell reductions higher than the limit of detection, however, the live cell counts by flow cytometry showed a 1.82 log reduction. Similar phenomena has been observed with Canadian honeys tested on log phase *E. coli* which, by flow cytometry, resulted in 26 to 7.8% of injured cells and above 40% of live cells which did not grow once plated (Brudzynski and Sjaarda, 2014). This dissimilarity between culturable and flow cytometry counts is possibly due to the presence of viable but non-culturable cells (Cerca et al., 2011) which may be a consequence of: (i) during the first stages of phage infection, cells must remain viable to replicate new phage progeny and therefore are detected in the SYTO^+^/PI^-^ quadrant, however, once plated these will not form colonies; (ii) when honey is applied, the minor perturbations and shrinkage observed in cells might also affect their cultivability.

Some authors describe honeys bacteriostatic effect on bacteria, not allowing bacterial growth up to even 4 days (reviewed in Molan, 1992). Our SEM analysis on *E. coli* biofilm cells (**Figure 5**) show cell collapse and leakage of the cytoplasm. It is known that bacterial survival requires integral membrane architecture so that transmembrane potential can be regulated, as these are essential requirements for growth as well as cell metabolic activity. Thus, honeys action on *E. coli* biofilm cells makes them completely unable to resume re-growth, thus contradicting the bacteriostatic assumption. Besides this work, to our knowledge, cytoplasmic leakage due to honey has only been reported toward *S. aureus* using honey from two stingless bees (Nishio et al., 2016). The effect of MGO on *E. coli* and *Bacillus subtilis* has been studied previously by other authors who observed that with concentrations above 1 mM, both species presented fewer fimbriae and flagella (Rabie et al., 2016). Additionally, MGO alone has been shown to cause membrane damage and shrinkage similar to the observations we report herein using PF2 honey. Studies with MGO have also addressed *Proteus mirabilis* showing that this compound was able to diffuse through an established biofilm matrix and effectively kill bacterial cells (Majtan et al., 2014). This suggests that in our samples the MGO content can be responsible for shrinkage and membrane damage. Spheroplasts formation in *E. coli* was induced by a buckwheat honey and these spheroplasts were highly prone to lysis (Brudzynski and Sjaarda, 2014). The perturbations that honey caused in *E. coli* surface included several phenomena that have been also described using antibiotics and also antimicrobial peptides (Alves et al., 2010; Prince et al., 2016). For instance, the antimicrobial peptides BP100, a short cecropin A-melittin hybrid peptide, and pepR, an antimicrobial peptide derived from the dengue virus, caused all membrane deformations as those we observed with honey (Alves et al., 2010). Nisin on the other hand, a polycyclic antibacterial peptide and class I pore-forming bacteriocin, forms pores that cause rapid dissipation of transmembrane electrostatic potential resulting in membrane permeabilization and rapid bacterial cell death. Electron microscopy of nisin-treated *E. coli* showed, similarly to our results, minor changes in the cell size (Prince et al., 2016), however, no cell collapse or cytoplasmic leakage. The minor perturbations of the bacterial envelope, shrinkage and collapse were more common in cells imaged shortly after the beginning of the honey treatment which points to an interaction of honey with the negatively charged lipopolysaccharide (LPS) outer layer of *E. coli* cells (**Figure 5**).

Several of these described effects on cell structure that alter cell viability were corroborated and reinforced by TEM images. In fact, the cell envelope shrinkage observed by TEM in all samples treated with phage (**Figures 6d–f**) seemed to be a similar phenomenon to the described by SEM, that leaded subsequently to the disruption of the cell wall and to the release of cellular content (**Figures 5j–o**). The activity of both antimicrobials together is evidenced in these images. Besides, the perturbations in the cell surface caused by honey as visualized by SEM, detachment of the cytoplasmic membranes and cytoplasm condensation were revealed by TEM. The occurrence of clear zones in the central or apical zones of cells may be inducing their collapse (**Figures 5b–i**). The antimicrobial observations are not unique for honey. For instance, the antimicrobial peptide arenicin shows similar effects in cells—it causes membrane blebs formation, release and condensation of cytoplasmic material, and detachment of the outer membrane from the plasma membrane (Andrä et al., 2008).

The combinatorial effect of phage and honey revealed that phage–PF2_25%_ displayed higher antibiofilm activity than honey alone throughout all the experiment, indicating that the phage action was determinant in enhancing honey’s action. Not surprisingly, EC3a phage remained active and highly concentrated for 24 h in PF2_25%_. However, more than an additive effect, PF2_25%_ together with phage EC3a had a synergic influence at both 12 and 24 h. This is a totally new outcome corroborated by flow cytometry experiments showing that the simultaneous application stemmed the progress of the *E. coli* biofilms with phages targeting the cells with highest SYTO uptakes that PF2 honey failed to address. According to the SEM observations, the use of phage–PF2 honey combination caused some biofilm cells to be subject of phage attack and others to be damaged and killed by honey. Until 4 h of phage exposure to U3_25%_ the loss in viability was small suggesting that progeny EC3a phages have enough time to reach and infect neighbor cells. Nonetheless, no synergic effect of phage with this honey was noticed. Therefore, synergy using phage and honey can be honey and phage dependent. Coupling phages to high honey concentrations [50% (w/v)] had no advantages since honey alone destroyed already the vast majority of biofilm cells. This can be a consequence of the higher antiviral activity of this honey concentration. Nevertheless, an advantage of the treatment combining phages with honey is the lower emergence of phage insensitive mutants.

Cell surface charge is highly dependent on both the composition of the surface and the nature of the surrounding medium (Soon et al., 2011). Herein, we used zeta potential to analyze cell surface charge. *E. coli* biofilm cells had always negative zeta potential values (Supplementary Figure S1) without any background conductivity alteration due to honey and/or phages. The conductivity and pH are known to interfere in the adsorption of ions onto bacterial cells and in the degree of ionization of charged moieties on the cell surface (Hong and Brown, 2008), however, this is not applicable to our study since these conditions did not vary considerably. The dense negatively charged barrier in Gram-negative bacteria is mostly a result of several forces which are needed to stabilize divalent cations that bind LPS molecules within the membrane (Peterson et al., 1985). Besides LPS, the primary component of the outer leaflet, the negative zeta potential of Gram-negative bacteria is also dictated by the presence of a high number of fimbriae in the cell morphology, capsule (Bayer and Sloyer, 1990; Soon et al., 2011), and even the growth state of bacteria (Soon et al., 2011). Our results show that honey treatment of biofilms led to cells with less negative zeta potential values. The corresponding rise in zeta potential of honey-treated biofilms is most plausibly explained by the alterations that occurred in the cell morphology that were visible by SEM in the *E. coli* outer membrane. This is in agreement with some results where different antibiotics led to less negatively charged zeta potential in Gram-negative bacteria as a consequence of modification or complete loss of LPS and/or lipid A from the outer membrane (Winfield et al., 2005; Gooderham and Hancock, 2009; Moffatt et al., 2010). Zeta potential values after 24 h treatment with phage and phage–honey [50% (w/v)] were more negative. The difference in the phage-alone 24 h treatment can be credited to the poor effectiveness of the treatment at this time point that led to a high number of cells, insensitive to phage, surviving and continuing their growth. These cells are most likely at the exponential phase and this corroborates a few studies (Eboigbodin et al., 2006; Bolster et al., 2010), where exponentially growing cells had more negative zeta potential than stationary phase cells. However, the phenomenon leading to a more negative zeta potential value after phage–honey [50% (w/v)] are unknown and warrant further investigation.

Summarizing, our results indicate that the activity of honey at 25% (w/v) is enhanced by the addition of phages and the resulting antimicrobial effect is similar to 50% (w/v) honey applied to 24- and 48-h-old biofilms. This result reinforces the potential use of both antimicrobials together, taking advantage of honey’s antibiofilm activity, and of the phages ability to lyse specific bacteria. Additionally, microbial resistance to honey has never been reported, and their antiviral effect is advantageous compared to phage therapy alone, reducing the emergence of phage insensitive mutants. The use of a more diluted honey solution is known to be advantageous, not only due to a potential lower cost of treatment, but also since a more liquid solution might be therapeutically more desirable as a topical rinsing solution maximizing the tolerability and practicality of the delivery technique. The pioneering combined delivery of phage and honey is thus a promising antimicrobial alternative, particularly in the treatment of chronic wound infections with *E. coli* and can be an option also for other species that do not respond to antibiotic therapy.

## Author Contributions

AO, AH, and SS conceived the study. AO, HR, LM, AH, and SS analyzed data. HR, AS, MS, JS, LM, and CR performed the experiments. AO and HR wrote the paper. All authors critically analyzed and revised the manuscript.

## Conflict of Interest Statement

The authors declare that the research was conducted in the absence of any commercial or financial relationships that could be construed as a potential conflict of interest.
