# Supplementary material for: Synergistic Antimicrobial Interaction between Honey and Phage against Escherichia coli Biofilms
Source: Front Microbiol. 2017 Dec 8;8:2407. doi: 10.3389/fmicb.2017.02407 (PMC5727068; doi:10.3389/fmicb.2017.02407)
Supplement: Supplementary file 1 [file Table_1.pdf]

Table S1 - Summary of the characteristics of honeys tested in this work

	Portuguese Honeys													Commercial Manuka
Honey identification*	Ericaceae – U3	Ericaceae – U2	Ericaceae – U1	Prunus – P1	Polyflora - PF1	Eucalyptus – E2	Polyflora – PF4	Lavandula L2	Lavandula - L1	Polyflora – PF2	Eucalyptus – E1	Polyflora – PF3	Lavandula - L3	
Colour	Dark ambar	Extra Ligth Amber	Ligth Amber	White	Extra White	Extra White	Extra Ligth Amber	Extra White	Extra White	Ligth Amber	White	Extra Ligth Amber	White	Ligth Amber
pH	**4.04**	4.32	4.12	4.42	3.15	4.20	4.55	3.34	3.88	**4.13**	3.70	4.42	3.60	3.50
Protein ($mg.g^{-1}$)	**81.7**	10.8	75.3	55.9	24.2	25.7	19.1	8.9	49.2	**16.6**	31.5	45.6	56.5	60.9
HMF ($mg.kg^{-1}$)	**204.6**	151.9	745.5	50.1	77.1	65.9	73.4	73.4	191.6	**47.9**	77.1	45.7	115.3	189.4
MGO (mg.L) 100% honey	**2092.4**	1266.4	989.0	123.9	184.2	77.8	47.5	32.1	31.5	**316.6**	135.4	39.5	155.4	756.5
MIC *Escherichia coli CECT* 434	**25%**	25%	25%	25%	25%	25%	25%	25%	25%	**12.5%**	12.5%	12.5%	na	12.5%
MIC *Escherichia coli* K12 JM109	**25%**	25%	25%	25%	25%	25%	25%	25%	25%	**25%**	25%	25%	na	
MIC *Pseudomonas aeruginosa* PAO1 (DSM 22644)	**25%**	25%	25%	25%	25%	25%	25%	25%	25%	**12.5%**	12.5%	25%	na	25%
MIC *Acinetobacter pittii*-CEB-Ap[a)]	**12.5%**	na	25%	12.5%	25%	na	25%	12.5%	25%	**6.25%**	6.25%	12.5%	na	
MIC *Staphylococcus aureus* ATCC 19685	**25%**	na	25%	12.5%	50%	na	12.5%	12.5%	50%	**6.25%**	3.125%	12.5%	na	12.5%
Conductivity (µS/cm)	**622**									**753**				

* honeys were identified according to the information given by the regional beekeepers of the predominant floral source in the area. [a)] clinical isolate (Oliveira et al., 2017). na - not analysed.
