# Supplementary material for: Synergistic Antimicrobial Interaction between Honey and Phage against Escherichia coli Biofilms
Source: Front Microbiol. 2017 Dec 8;8:2407. doi: 10.3389/fmicb.2017.02407 (PMC5727068; doi:10.3389/fmicb.2017.02407)
Supplement: Supplementary file 2 [file Table_2.pdf]

Table S2 – Pollen analysis of PF2 and U3 honeys

	**PF2**	**U3**
**Predominant pollen (> 45%)**	***Castanea sativa* (56%)**	
Secondary pollen (16-45%)	*Eucalyptus* spp. (26%)	*Erica* spp. (30%)<br>*Rubus* spp. / *Eriobotrya* spp. (21%)<br>*Castanea sativa* (18%)
Minoritary pollen (3-15%)	*Rubus* spp. / *Eriobotrya* spp. (10%)<br>*Raphanus raphanistrum* (4%)	*Eucalyptus* spp. (16%)<br>*Raphanus raphanistrum* (8%)
Identified pollen (<3%)	*Orlaya daucoides* (1%)<br>*Lotus creticus* (1%)<br>*Prunus* spp. / *Pyrus* spp. (1%)<br>*Lithodora fruticosa* (0%)<br>*Hypericum perforatum* (0%)<br>*Lycium* spp. (0%)<br>*Scrophularia canina* (0%)	*Cytisus* spp. (2%)<br>*Echium plantagineum* (1%)<br>*Capsella bursa-pastoris* (1%)<br>*Lotus creticus* (1%)<br>*Helianthus annus* (0%)<br>*Hypecoum* spp. (0%)<br>*Crataegus monogyna* (0%)
Nectarless pollen	*Cistaceae*<br>*Papaverrhoeas*	*Quercus* spp.<br>*Cistaceae*<br>*Ulex* spp.
