# Supplementary material for: Synergistic Antimicrobial Interaction between Honey and Phage against Escherichia coli Biofilms
Source: Front Microbiol. 2017 Dec 8;8:2407. doi: 10.3389/fmicb.2017.02407 (PMC5727068; doi:10.3389/fmicb.2017.02407)
Supplement: Supplementary file 3 [file Table_3.pdf]

Table S3 - Features of phage EC3a open reading frames and their homology to other phage proteins

DNA				Protein						
ORF	Start	End	Length	Mw	pI	aa	Function	Homolog	E-value	Motifs
1	414	893	480	17.63	8.78	159	hypothetical protein	hypothetical protein [Escherichia phage e4/1c]	8.00E-20	
1A	893	1003	111	4.10	9.12	36	hypothetical protein	hypothetical protein Rogue1_0066 [Enterobacteria phage vB_EcoS_Rogue1]	4.00E-07	
2	1079	1414	336	12.70	9.56	111	hypothetical protein	hypothetical protein JK_46 [Escherichia phage Jk06]	6.00E-25	
3	1485	1685	201	7.26	3.79	66	hypothetical protein	hypothetical protein JK_45 [Escherichia phage Jk06]	3.00E-20	
4	1699	1917	219	7.77	9.80	72	hypothetical membrane protein			1 TMD
5	2111	2371	261	9.71	8.46	86	hypothetical protein	hypothetical protein rtp5 [Escherichia phage Rtp]	2.00E-49	
6	2441	2671	231	8.63	4.42	76	hypothetical protein	hypothetical protein ACG-M12_0005 [Enterobacteria phage vB_EcoS_ACG-M12]	9.00E-27	
7	2668	2994	327	12.28	4.47	108	hypothetical protein	hypothetical protein SP36_2 [Salmonella phage 36]	5.00E-33	
8	3337	3516	180	6.91	4.95	59	hypothetical protein	hypothetical protein rtp11 [Escherichia phage Rtp]	4.00E-32	
9	3568	3693	126	5.02	4.19	41	hypothetical protein	hypothetical protein rtp12 [Escherichia phage Rtp]	1.00E-16	
10	3690	3833	144	5.78	9.64	47	hypothetical protein	hypothetical protein rtp13 [Escherichia phage Rtp]	1.00E-25	

11	3904	4044	141	5.55	3.84	46	hypothetical protein	hypothetical protein ACG-M12_0011 [Enterobacteria phage vB_EcoS_ACG-M12]	7.00E-07	
12	4044	4283	240	9.12	9.89	79	hypothetical protein	hypothetical protein ACG-M12_0012 [Enterobacteria phage vB_EcoS_ACG-M12]	7.00E-52	
13	4311	4445	135	4.94	4.06	44	hypothetical membrane protein			1 TMD
15	4863	5045	183	6.66	8.22	60	hypothetical protein	hypothetical protein ACG-M12_0013 [Enterobacteria phage vB_EcoS_ACG-M12]	3.00E-35	
16	5157	5663	507	18.75	5.82	168	terminase. small subunit	putative terminase small subunit [Escherichia phage Rtp]	6.00E-114	PF16677 GP3_package
17	5681	7246	1566	59.38	6.15	521	terminase. large subunit	putative terminase large subunit [Enterobacteria phage vB_EcoS_ACG-M12]	0	PF03237 Terminase_6
18	7316	7648	333	12.50	4.73	110	hypothetical protein	hypothetical protein ACG-M12_0016 [Enterobacteria phage vB_EcoS_ACG-M12]	1.00E-35	
19	7706	8974	1269	46.99	4.56	422	portal protein	putative portal protein [Enterobacteria phage vB_EcoS_ACG-M12]	0	PF06381 DUF1073
20	8955	10043	1089	39.29	4.74	362	prohead protease	putative prohead protease [Enterobacteria phage vB_EcoS_ACG-M12]	0	PF09979 DUF2213
21	10056	10568	513	16.95	6.24	170	hypothetical protein	gp54 [Escherichia phage EB49]	1.00E-95	
22	10692	11636	945	34.42	5.02	314	major capsid protein	hypothetical protein ACG-M12_0020 [Enterobacteria phage vB_EcoS_ACG-M12]	0	PF09950 DUF2184

23	11729	11974	246	9.06	8.20	81	hypothetical protein	hypothetical protein ACG-M12_0021 [Enterobacteria phage vB_EcoS_ACG-M12]	8.00E-50	
24	12016	12417	402	14.34	7.18	133	hypothetical protein	Halo29 [Escherichia phage RES-2009a]	7.00E-91	PF13262 DUF4054
25	12414	12785	372	13.71	5.19	123	hypothetical protein	Halo30 [Escherichia phage RES-2009a]	9.00E-80	
26	12778	13215	438	15.75	9.36	145	hypothetical protein	hypothetical protein ACG-M12_0024 [Enterobacteria phage vB_EcoS_ACG-M12]	2.00E-100	
27	13205	13606	402	15.13	5.87	133	hypothetical protein	hypothetical protein ACG-M12_0025 [Enterobacteria phage vB_EcoS_ACG-M12]	3.00E-92	PF13554 DUF4128
28	13622	14278	657	23.36	4.58	218	major tail protein	putative major tail protein [Enterobacteria phage vB_EcoS_ACG-M12]	2.00E-149	PF08813 Phage_tail_3
29	14389	14637	249	8.90	9.17	82	hypothetical protein	hypothetical protein ACG-M12_0027 [Enterobacteria phage vB_EcoS_ACG-M12]	2.00E-17	1 SP
30	14677	14991	315	11.80	4.45	104	tail assembly chaperone	hypothetical protein ACG-M12_0028 [Enterobacteria phage vB_EcoS_ACG-M12]	8.00E-67	PF08748 Phage_TAC_4
31	15000	15311	312	12.07	4.01	103	hypothetical protein	hypothetical protein ACG-M12_0029 [Enterobacteria phage vB_EcoS_ACG-M12]	3.00E-68	PF08809 DUF1799
32	15347	18325	2979	106.97	5.47	992	tail tape measure protein	putative tail tape-measure protein [Enterobacteria phage vB_EcoS_ACG-M12]	0	PF06791 TMP_2; PF09718 Tape_meas_lam_C
33	18356	18706	351	13.34	7.25	116	minor tail protein	putative minor tail protein [Escherichia phage Rtp]	7.00E-74	PF05939 Phage_min_tail

34	18745	19500	756	27.88	4.81	251	minor tail protein	putative minor tail protein [Enterobacteria phage vB_EcoS_ACG-M12]	0	PF05100 Phage_tail_L
35	19511	20269	759	29.33	5.55	252	minor tail protein	putative minor tail protein [Enterobacteria phage vB_EcoS_ACG-M12]	0	PF14464 Prok-JAB; PF00877 NLPC_P60
36	20250	20822	573	20.09	9.98	190	tail assembly protein	putative tail assembly protein [Enterobacteria phage vB_EcoS_ACG-M12]	4.00E-135	PF06805 Lambda_tail_I; 1 TMD
37	20873	24289	3417	125.85	4.49	1138	tail fiber protein	putative tail fiber protein [Enterobacteria phage vB_EcoS_ACG-M12]	0	PF13550 Phage-tail_3; PF09327 DUF1983
38	24320	25282	963	33.85	5.92	320	hypothetical protein	hypothetical protein ACG-M12_0038 [Enterobacteria phage vB_EcoS_ACG-M12]	0	
39	25282	25524	243	8.18	8.50	80	lipoprotein	putative lipoprotein [Escherichia phage bV_EcoS_AHS24]	6.00E-42	
40	25604	26251	648	25.16	5.24	215	DNA-binding protein	phage regulatory protein Rha [Pseudomonas phage PS-1]	8.00E-22	PF09669 Phage_pRha
41	26617	27048	432	16.19	7.18	143	hypothetical protein	hypothetical protein rtp46 [Escherichia phage Rtp]	1.00E-67	
42	27052	28017	966	36.21	5.18	321	exodeoxyribonuclease VIII	putative exodeoxyribonuclease VIII [Enterobacteria phage vB_EcoS_ACG-M12]	0	PF12684 DUF3799
43	28091	28741	651	23.80	5.03	216	recombination protein	putative recombination protein [Enterobacteria phage vB_EcoS_ACG-M12]	6.00E-152	PF04404 ERF
44	28786	29217	432	16.70	7.67	143	ssDNA binding protein	putative single-stranded DNA binding protein [Escherichia phage Rtp]	5.00E-73	

45	29244	32054	2811	101.08	4.97	936	tail fiber protein	putative tail fiber protein [Enterobacteria phage vB_EcoS_ACG-M12]	0	
46	32142	33065	924	34.65	5.67	307	DNA primase	putative DNA primase [Enterobacteria phage vB_EcoS_ACG-M12]	3.00E-169	PF08273 Prim_Zn_Ribbon
47	33062	33541	480	18.53	9.74	159	HNH-endonuclease	HNH-endonuclease [Vibrio phage pYD38-A]	3.00E-41	PF13392 HNH_3
48	33594	34067	474	18.03	10.99	157	transcriptional regulator	putative transcriptional regulator [Escherichia phage Rtp]	8.00E-112	
49	34169	36163	1995	75.14	8.06	664	ATP-dependent helicase	putative ATP-dependent helicase [Escherichia phage Rtp]	0	PF00271 Helicase_C
50	36165	36584	420	15.64	7.91	139	hypothetical protein	hypothetical protein rtp54 [Escherichia phage Rtp]	4.00E-93	PF08774 VRR_NUC
51	36663	36857	195	7.33	4.01	64	hypothetical protein	hypothetical protein JK_68 [Escherichia phage Jk06]	3.00E-35	
52	36857	37078	222	8.96	3.99	73	hypothetical protein	hypothetical protein ACG-M12_0054 [Enterobacteria phage vB_EcoS_ACG-M12]	4.00E-32	
53	37078	37203	126	4.72	4.77	41	hypothetical protein	hypothetical protein ACG-M12_0057 [Enterobacteria phage vB_EcoS_ACG-M12]	9.00E-18	
54	37200	37448	249	9.50	9.95	82	hypothetical protein	hypothetical protein ACG-M12_0058 [Enterobacteria phage vB_EcoS_ACG-M12]	2.00E-53	
55	37451	37702	252	9.40	4.28	83	hypothetical protein	hypothetical protein rtp60 [Escherichia phage Rtp]	2.00E-53	
56	37786	38916	1131	42.00	7.03	376	hypothetical protein	hypothetical protein AHP42_45 [Escherichia phage bV_EcoS_AHP42]	0	

57	39001	39177	177	6.65	4.14	58	hypothetical protein	hypothetical protein ACG-M12_0064 [Enterobacteria phage vB_EcoS_ACG-M12]	9.00E-35	
58	39309	39524	216	7.44	10.83	71	holin	putative holin [Enterobacteria phage vB_EcoS_ACG-M12]	1.00E-40	1 TMD
59	39525	40010	486	17.52	10.07	161	endolysin	putative endolysin [Escherichia phage Rtp]	5.00E-98	PF00959 Phage_lysozyme; 1 SP
60	39986	40375	390	13.17	9.76	129	hypothetical membrane protein	hypothetical protein rtp65 [Escherichia phage Rtp]	1.00E-72	1 TMD; 1 SP
61	40390	40725	336	12.81	8.32	111	hypothetical protein	hypothetical protein ACG-M12_0068 [Enterobacteria phage vB_EcoS_ACG-M12]	2.00E-67	
62	40729	42312	1584	58.18	6.34	527	hypothetical protein	hypothetical protein rtp67 [Escherichia phage Rtp]	0	PF13148 DUF3987
63	42387	42806	420	16.27	9.67	139	hypothetical protein	hypothetical protein rtp69 [Escherichia phage Rtp]	3.00E-63	
64	42806	42967	162	6.30	10.40	53	hypothetical protein	hypothetical protein rtp70 [Escherichia phage Rtp]	7.00E-29	
65	42967	43146	180	6.98	3.73	59	hypothetical protein			
66	43203	43364	162	6.05	4.17	53	hypothetical protein	hypothetical protein ACG-M12_0072 [Enterobacteria phage vB_EcoS_ACG-M12]	2.00E-30	
67	43364	43603	240	8.85	9.26	79	hypothetical protein	hypothetical protein rtp73 [Escherichia phage Rtp]	1.00E-48	
68	43600	43788	189	7.09	4.06	62	hypothetical protein	hypothetical protein ACG-M12_0074 [Enterobacteria phage vB_EcoS_ACG-M12]	9.00E-06	
69	43799	43978	180	6.69	3.96	59	hypothetical protein	hypothetical protein kp_75 [Enterobacterio phage phiKP26]	2.00E-29	

70	44017	44133	117	4.37	8.51	38	hypothetical membrane protein	1 TMD

*TMD - Transmembrane domain; SP - Signal peptide; PF – Pfam
