# Supplementary material for: Synergistic Antimicrobial Interaction between Honey and Phage against Escherichia coli Biofilms
Source: Front Microbiol. 2017 Dec 8;8:2407. doi: 10.3389/fmicb.2017.02407 (PMC5727068; doi:10.3389/fmicb.2017.02407)
Supplement: Supplementary file 4 [file Table_4.pdf]

Table S4 - Evaluation of the effect of PF2 and U3 honeys. EC3a phage and their combination on *E. coli* biofilms using culturable cell counting and flow cytometry analysis.

	Flow cytometry analysis				CFU plating	
**Condition**	**SYTO BC MFI ± SD**	**PI MFI ± SD**	**Live cells (log_{10} cells.mL^{-1}) ± SD**	**Treatment – control (log_{10} cells.mL^{-1}) ± SD**	**Culturable cells (log_{10} CFU.mL^{-1}) ± SD**	**Treatment – control (log_{10} cells.mL^{-1}) ± SD**
**Control**	754 ± 8	153 ± 14	8.68 ± 0.12	0	7.85 ± 0.54	0
**$PF2_{25\%}$**	2552 ± 478	197 ± 7	8.53 ± 0.02	0.15 ± 0.12	4.25 ± 0.32	3.60 ± 0.46
**$U3_{25\%}$**	2525 ± 203	298 ± 3	8.27 ± 0.04	0.41 ± 0.12	5.91 ± 0.71	1.94 ± 0.77
**$U3_{50\%}$**	1666 ± 61	193 ± 8	7.07 ± 0.03	1.61 ± 0.12	5.39 ± 0.73	2.46 ± 0.80
**EC3a**	4126 ± 214	356 ± 58	7.16 ± 0.00	1.52 ± 0.12	6.64 ± 0.27	1.21 ± 0.40
**$PF2_{25\%}$ + EC3a**	1659 ± 98	227 ± 9	6.86 ± 0.04	1.82 ± 0.12	LOD*	>5.85
**$U3_{25\%}$ + EC3a**	4857 ± 634	234 ± 28	7.89 ± 0.01	0.79 ± 0.12	3.83 ± 0.63	4.02 ± 0.71
**$U3_{50\%}$ + EC3a**	1121 ± 146	163 ± 3	7.39 ± 0.04	1.29 ± 0.12	4.29 ± 0.18	3.56 ± 0.21

*Below the limit of detection (LOD)
