# Supplementary figures and images for: Synergistic Antimicrobial Interaction between Honey and Phage against Escherichia coli Biofilms
Source: Front Microbiol. 2017 Dec 8;8:2407. doi: 10.3389/fmicb.2017.02407 (PMC5727068; doi:10.3389/fmicb.2017.02407)

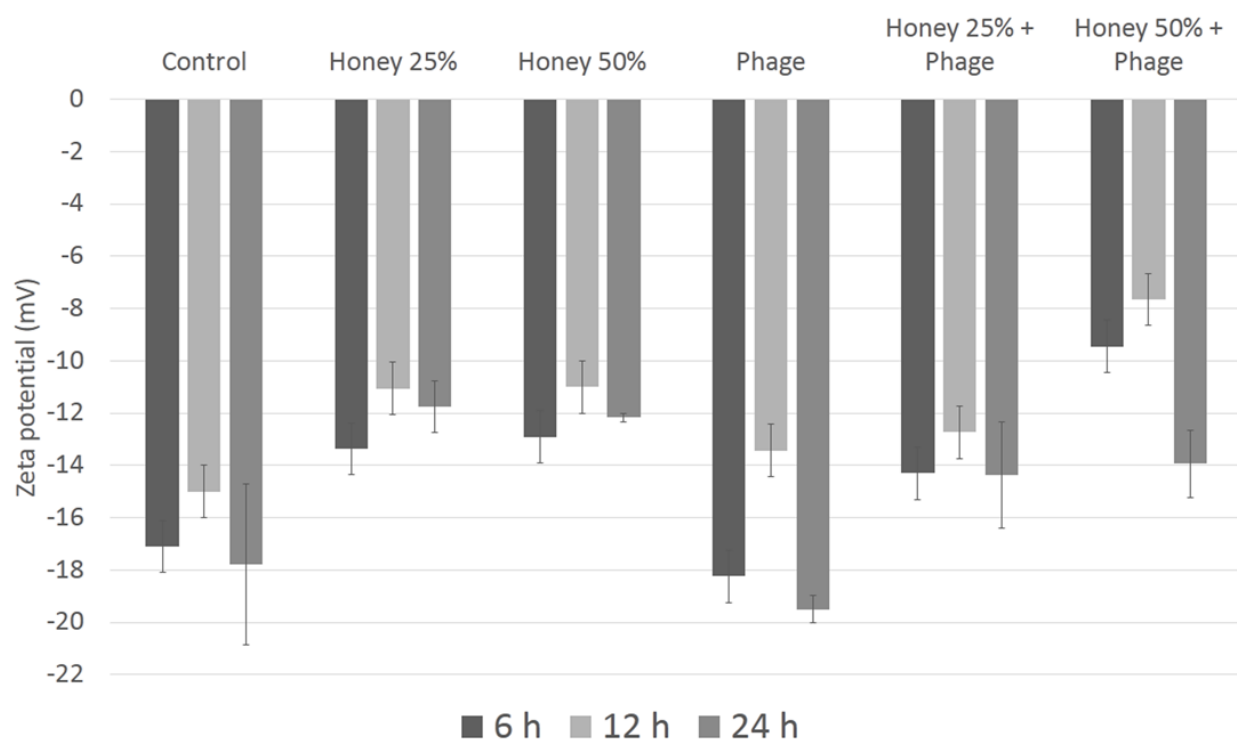

Figure S1 – Zeta potential of *E. coli* biofilm cells after the different treatments.

Supplement: Supplementary file 5 [file Image_1.pdf]
